# Blood acid–base, haematological and haemostatic effects of hydroxyethyl starch (130/0.4) compared to succinylated gelatin colloid infusions in normovolaemic dogs

**DOI:** 10.4102/jsava.v91i0.1990

**Published:** 2020-06-04

**Authors:** Roxanne K. Buck, Lynette Bester, Keagan J. Boustead, Abdur R. Kadwa, Gareth E. Zeiler

**Affiliations:** 1 Department of Companion Animal Clinical Studies, Faculty of Veterinary Science, University of Pretoria, Onderstepoort, South Africa; 2 Companion Animal Health Centre, School of Animal and Veterinary Science, University of Adelaide, Adelaide, Australia; 3 Section of Anaesthesia and Critical Care, Valley Farm Animal Hospital, Pretoria, South Africa

**Keywords:** acid–base balance, coagulation, Gelofusine, synthetic colloids, Voluven, full blood count (FBC), complete blood count (CBC)

## Abstract

Synthetic colloids are commonly administered to dogs to treat absolute or relative hypovolaemia. Voluven^®^ (tetrastarch 130/0.4) and Gelofusine^®^ (succinylated gelatin) are available to veterinarians in South Africa. In humans, use of these products has caused acid–base derangements, changes in haematology and impaired haemostasis. We aimed to investigate these effects in healthy normovolaemic dogs. Eight healthy adult beagle dogs underwent a cross-over study, receiving Voluven^®^ or Gelofusine^®^ (10 mL/kg/h for 120 min) once each with a 14-day washout between treatments. Dogs were premedicated with dexmedetomidine (10 *µ*g/kg intramuscularly). Anaesthesia was induced with propofol and the dogs were maintained with isoflurane-in-oxygen. The anaesthetised dogs were connected to a multi-parameter monitor to monitor physiological parameters throughout. Catheters placed in a jugular vein and dorsal metatarsal artery allowed sampling of venous and arterial blood. Blood was collected immediately prior to commencement of colloid infusion, after 60 min infusion and at the end of infusion (120 min) to allow for arterial blood gas analysis, haematology and coagulation testing (activated partial thromboplastin time [aPTT], prothrombin time [PT] and thromboelastography [TEG]). There was no effect, between treatments or over time, on blood pH. The haemoglobin concentration, erythrocyte count and haematocrit decreased significantly over time (all *p* < 0.01), with no differences between treatments, and remained within normal clinical ranges. There were no differences between treatments or over time for the TEG, aPTT and PT tests of haemostasis. At the dose studied, Voluven^®^ and Gelofusine^®^ had comparably negligible effects on blood acid–base balance and coagulation in normovolaemic dogs.

## Introduction

Synthetic colloid-based fluids are indicated to resuscitate the intravascular compartment during states of relative or absolute hypovolaemia. These fluids have been also used for acute normovolaemic haemodilution and to prime vascular catheters, circuits and pump units required for extracorporeal oxygenation and cardiopulmonary bypass.

The available synthetic colloid formulations are derived from hydroxyethyl starches (HES), modified fluid gelatin (MFG) and dextrans. The colloid component is suspended in various crystalloids to create a number of proprietary products. In South Africa, HES-based (tetrastarch 130/0.4; Voluven^®^ [VOL]) and MFG-based (succinylated gelatin; Gelofusine^®^ [GEL]) products are readily available to veterinarians. Both these fluids are classified as being isotonic and isooncotic whereby the fluids have a similar theoretical osmolality and oncotic pressure, respectively, to that of human plasma.

The use of both HES- and MFG-based colloids has resulted in acid–base, haematological and haemostatic derangements in humans (Severs, Hoorn & Rookmaaker 2015). Although studies in animals subjected to trauma have suggested that the blood volume expansion produced by HES and MFG during resuscitation may be similar, direct comparisons between these fluids are not reported in dogs (Dubniks, Persson & Grände 2009). Furthermore, the effects of these fluids on acid–base, haematology and haemostasis are rarely reported in dogs.

The foremost concern regarding the use of synthetic colloids is the risk of developing a hyperchloraemic metabolic acidosis (Langer et al. 2015). The Stewart approach to acid–base evaluation is suited to determining acid–base changes that occur during administration of all intravenous fluids because it describes changes in pH with relation to changing plasma components (Muir 2017). This approach describes changes in hydrogen ion concentration (in effect, the pH) with respect to three independent factors: (1) the partial pressure of carbon dioxide (PCO_2_), (2) the concentration of weak acids (A_TOT_) and (3) the strong ion difference (SID), which is the mathematical difference between all strong cation and anion concentrations (Langer et al. 2014; Muir 2017). Fluids with an SID of 24 mEq/L are considered to be ‘balanced’ and minimally influence acid–base balance (Muir et al. 2011). The stereotypical ‘unbalanced’ fluid, physiological saline (0.9% NaCl) is the crystalloid fluid that suspends the tetrastarch of VOL (Langer et al. 2015). Physiological saline has an SID of 0 mEq/L and thus can produce a hyperchloraemic acidosis, creating a fear for development of acidosis following the use of VOL (Muir et al. 2011). However, larger volumes of 0.9% NaCl (in excess of 70 mL/kg) are required to produce a hyperchloraemic acidosis in dogs as compared to humans, where a bolus of 30 mL/kg produced a hyperchloraemic acidosis (West et al. 2013). The crystalloid fluid that suspends succinylated gelatin in GEL is ‘balanced’, with an SID of 34 mEq/L, making the development of acidosis less likely. Alterations in PCO_2_ and A_TOT_ are not considered major causes of acid–base derangements during HES and MFG administration.

The molecular modification of HES makes them resistant to degradation by serum α-amylase, thus contributing to their longevity within plasma. The concentration, molecular weight and C_2_/C_6_ ratio determine the rate of degradation by serum α-amylase. The degradation products are excreted via the kidneys, phagocytosed by the reticuloendothelial system or pinocytosed by a variety of cells. The cellular lysosomes do not have the ability to degrade HES, thus the molecules remain within these cells potentially adversely affecting them. Over the last decade, the use of HES in critically ill human patients has been associated with increased risk of acute kidney injury, increased rates of renal replacement therapy and hepatopathy, amongst others (Bae et al. 2017). Similarly, the use of pentastarch (10% HES 250/0.5/5:1) was associated with an increased incidence of acute kidney injury in dogs. Conversely, a retrospective cohort study comparing dogs treated with either a tetrastarch or crystalloids found no difference between the incidence of acute kidney injury or serum creatinine between groups (Sigrist, Kälin & Dreyfus 2017). However, Bae et al. (2017) published a case report wherein they describe the development of acute kidney injury in a dog after administration of a tetrastarch (HES 6% 130/0.4), although the animal in question already had underlying renal disease.

Haemoglobin is the major transporter of oxygen (Haldane effect) and also contributes to acid–base buffering of carbon dioxide (Bohr effect) within the plasma (Muir 2015). The importance of haemoglobin as an oxygen transporter is reflected in the equation used to calculate the oxygen content in 100 mL of arterial blood, as follows (Equation 1):

$$\begin{array}{l} {\text{CaO}}_{2}=({\text{SaO}}_{2}[\%]\times \text{Hb concentration }[\text{g}/\text{dL}]\times 1.34\;[{\text{mLO}}_{2}/\text{g}\\ \text{Hb}])+\left(0.003\;[\text{mL}/\text{mmHg of }{\text{O}}_{2}]\times {\text{PaO}}_{2}\;[\text{mmHg}]\right), \end{array}$$ [Eqn 1]

where CaO_2_ is the content of oxygen in the arterial blood; SaO_2_ is the saturation percentage of oxygen bound to haemoglobin; Hb concentration is the concentration of haemoglobin in 100 mL of blood; 1.34 is the Hüfner’s constant, which is the theoretical oxygen carrying capacity of haemoglobin, and PaO_2_ is the partial pressure of oxygen in the gaseous form saturated within the plasma (Haskins 2015). Therefore, a dilution of haemoglobin during synthetic colloid fluid administration could cause metabolic derangements reflected by hypoxaemia if the haemoglobin concentration decreases to less than 6.0 g/dL, the point at which anaerobic metabolism predominates (Jamnicki et al. 2003; Van der Linden et al. 1998). The haematological dilutional effect of VOL and GEL when administered to normovolaemic dogs is mostly unknown.

Voluven^®^ and GEL are known to cause coagulation derangements in humans and dogs. Voluven^®^ has been reported to cause hypocoagulability, whilst some studies have reported hypercoagulability following the use of GEL. The coagulopathies described are thought to result from dilutional and non-dilutional effects causing disruptions in primary (platelet plug formation) and secondary (fibrin clot formation) haemostasis. Dilutional effects are attributed to a decrease in concentration of circulating platelets and coagulation factors (De Jonge et al. 1998; Falco et al. 2012). The non-dilutional effects are thought to result from one of the following four possible mechanisms: (1) the binding of the colloid component of the fluids to platelets, altering their adhesive function (Franz et al. 2001; Phillips et al. 1988); (2) decrease in the function and activity of certain coagulation factors beyond simple dilution by unexplained mechanisms (De Jonge et al. 1998; Fenger-Eriksen et al. 2009; Gauthier et al. 2015; Kozek-Langenecker 2005); (3) colloid-induced hyperfibrinolysis (Gauthier et al. 2015) and (4) impaired fibrin polymerisation (De Jonge et al. 1998; Niemi et al. 2006).

Several *in vitro* and *in vivo* comparative studies have investigated the effects of VOL on haemostasis in dogs (Botto et al. 2018; Falco et al. 2012; Gauthier et al. 2015; Griego-Valles et al. 2017; McBride et al. 2013, 2016; Wurlod et al. 2015). Laboratory changes indicative of hypocoagulability at doses as low as 10 mL/kg – 20 mL/kg (corresponding to 1:10–1:5.5 dilutions *in vitro*) have been documented, although more significant changes are noted at doses above 30 mL/kg (1:4 dilution *in vitro*) (Falco et al. 2012; Griego-Valles et al. 2017; McBride et al. 2013, 2016). Hypocoagulable effects have been reported with *in vitro* use of GEL at dilutions as low as 10% – 20% (Brazil & Coats 2000; Egli et al. 1997). However, to the best of the authors’ knowledge, there have been no studies comparing the *in vivo* effects of GEL and VOL on haemostasis in dogs.

The aim of the present study was to describe and compare the acid–base, haematological and haemostatic effects of a fixed rate infusion of VOL or GEL administered intravenously for 120 minute to normovolaemic dogs. The outcome should allow for meaningful and clinically relevant conclusions regarding the safety and clinical effectiveness of these fluids in normovolaemic dogs.

## Materials and methods

### Animals and experimental design

The randomised crossover study was approved by the Animal Ethics Committee of the University of Pretoria (V039-17). An online randomiser generated the order (balanced single block design; www.randomization.com) of the dogs participating in the trial to receive two fluid therapies once each with a 14-day washout period.

Eight Beagle dogs with a mean (range) age of 5 (2–10) years and body mass of 12.2 (10.0–14.0) kg from the Onderstepoort Teaching Animal Unit (OTAU) were enrolled. The dogs were considered clinically healthy on the basis of a physical examination, haematocrit and total serum protein obtained 48 h before data collection.

Extensive haematological and biochemical profiling of the dogs was deemed unnecessary based on the work of Alef, Von Praun and Oechtering (2008). Their study concluded that haematological and biochemical testing was unnecessary in 84.1% of dogs after obtaining a history and performing a physical examination. Furthermore, only 8% of dogs were reclassified to a higher American Society of Anesthesiologists (ASA) risk score rating (Alef et al. 2008). In addition, haematological and biochemical changes in dogs under anaesthesia are well established (Alkattan & Helal 2013; Khurana et al. 2014; Wilson et al. 2004). One of the aims of the study was to determine these changes as the colloids were infused. It was therefore felt that utilising baseline variables before the animals were anaesthetised would unnecessarily skew the results.

The dogs were habituated to the hospital enclosures and routine husbandry procedures 2 weeks before the data collection was conducted. On the day before the data collection, the dogs were hospitalised and food, but not water, was withheld overnight.

### Experimental procedures

#### General anaesthesia and instrumentation

On the day of the procedures, the dogs were transferred to the theatre complex, weighed (floor scale) and allowed to rest in the cage for 30 min. The dogs were then premedicated with dexmedetomidine (0.01 mg/kg, intramuscularly [IM]; Zoetis). After 30 min, an indwelling intravenous (IV) catheter (20G Jelco; Smith and Nephew) was aseptically placed into a cephalic vein and secured. Anaesthesia was induced with Propofol (Propoven 1%; Fresenius Kabi) intravenously to effect, allowing orotracheal intubation. The Beagle dogs were placed into right lateral recumbency. General anaesthesia was maintained using isoflurane (Safeline) in 100% oxygen (fresh gas flow rate of 40 mL/kg/minute) delivered through a circle breathing system. A second indwelling intravenous catheter (18G Jelco) was aseptically placed into the jugular vein to allow for intermittent venous blood sampling. An arterial catheter (21G; Arrow International) was placed aseptically into the dorsal pedal artery to allow for direct blood pressure monitoring and intermittent arterial blood sampling.

A multi-parameter anaesthetic monitor (Cardiocap/5, Datex Ohmeda, GE Healthcare, United States [US]) was used to monitor physiological parameters. The side-stream gas sampler (sample rate 200 mL/min) was attached to the Y-piece to monitor end-tidal isoflurane concentration (FE′iso), carbon dioxide partial pressure (PE′CO_2_) and respiratory rate (*f*R).

Electrocardiograph (ECG) pads were placed on three paw pads (right arm [RA], left arm [LA] and left leg [LL]) and attached to the ECG leads to monitor the electrical activity of the heart and determine the heart rate (HR). A transmission pulse oximeter probe (SpO_2_) was placed on the tongue and an oesophageal temperature probe was inserted into the oesophagus to the level of the scapula. Direct arterial blood pressure was measured using an electronic transducer (BD DTX, Becton & Dickson Medical, NY, US) that was zeroed to atmospheric pressure at the level of the right atrium. Lastly, the cephalic catheter was flushed to ensure patency and then a primed administration set delivering the colloid fluid was attached to the catheter. Thereafter, the randomly allocated fluid, either VOL (Voluven^®^ 6%, Fresenius Kabi) or GEL (Gelofusine^®^ 4%, B. Braun Medical), was infused using an electronic infusion pump (Infusomat, B. Braun, Hessen, Germany) at a fixed rate of 10 mL/kg/h for a total of 120 min (total dose: 20 mL/kg). After 120 min, the colloid infusion was stopped, all monitoring devices and catheters, except the cephalic catheter, were removed and the dogs were allowed to recover in a heated and quiet recovery room. The dogs were monitored closely for 4 h after recovery for any untoward effects (allergic reactions or volume overload). Thereafter, the cephalic catheter was removed, and the dogs were returned into the care of the OTAU personnel for further observation and continuation of their normal daily routine. The dogs were rested for 14 days post-experimental procedures, where they were excluded from undergraduate student training activities.

#### Data collection

Baseline data were collected immediately prior to commencement of the colloid fluid infusion (T0), after which the infusion commenced and continued for 120 min. Throughout the infusion, physiological variables were recorded at 15-min intervals. Blood was collected at T0 (venous and arterial blood), at 60 min (T60; arterial blood only) and immediately after the end of the colloid fluid infusion (T120; venous and arterial blood). Two millilitres of blood were withdrawn into a 3-mL syringe from the respective catheters as a waste sample, which was discarded, prior to withdrawing blood samples.

A 2-mL arterial blood sample was obtained from the arterial catheter using a pre-heparinised syringe (BD A-Line, Becton Dickinson Ltd, Woodmead, RSA). Acid–base effects were determined by analysing the arterial blood sample within 2 min of collection using a daily calibrated, bench-top blood gas analyser (Siemens RAPIDPoint 500, Siemens Healthineers, Erlangen, Germany).

Venous blood (collected into a 10-mL syringe and injected into different vacuum storage tubes, which were inverted gently four to six times to allow for adequate mixing of blood with tube substances) was obtained from the jugular catheter. For determining the haematological effects, a 2-mL venous blood sample was collected in an Ethylenediaminetetraacetic acid (EDTA) tube, from which a differential cell count was determined using a daily calibrated cytometer (Advia 2120, Siemens Healthineers, Erlangen, Germany). For determining haemostatic effects, a 4-mL venous blood sample was collected in a citrate tube. Thromboelastography (TEG), activated partial thromboplastin time (aPTT) and prothrombin time (PT) were determined using daily calibrated machines (Haemoscope TEG 5000 Thrombelastograph Hemostasis Analyser, Haemonetics, Braintree, MA, US for TEG, and ACL Elite, Werfen, Barcelona, Spain for aPTT and PT). Experienced veterinary technologists analysed all the blood samples as per industry and machine manufacturer recommendations.

The arterial pulse pressure was calculated, for each time point per dog, as the mathematical difference between the systolic and diastolic arterial blood pressure values.

### Statistical analysis

The data were tested for normality by plotting histograms, evaluating descriptive statistics and performing the Anderson–Darling test for normality. Data were described using mean (standard deviation and range). Serial data (physiological, acid–base, haematological and coagulation variables) were compared between treatments over time using a general linear mixed model (fixed effect: treatment and time; random effect: beagle; interactions: treatment, time and treatment × time). *Post hoc* multiple comparisons were performed using Dunnett’s methods (control sample: baseline readings). Model fit was determined by visually inspecting the residual plots to assess linearity, homogeneity of variances, normality and outliers. Data were analysed using commercially available software (MiniTab) and results were interpreted at the 5% level (*p* < 0.05) of significance.

### Ethical considerations

The randomised crossover study was approved by the Animal Ethics Committee of the University of Pretoria (V039-17).

## Results

All dogs completed the study without untoward effects from the experimental procedures. The measured physiological parameters were mostly unremarkable, especially the heart rate, arterial blood pressures and pulse pressures and considered within normal expected ranges for an anaesthetised healthy dog within their weight group (Table 1). However, although not significant between treatments, there was a significant increase in respiratory rate, especially with the GEL treatment, over time (*p* < 0.01). There were significant differences between treatments for oesophageal temperature (*p* = 0.01), where GEL treatment had a higher normothermic temperature compared to VOL dogs, especially evident from 60 min onwards. Also, the SpO_2_ was lower, but remained above 93% oxygen–haemoglobin saturation, in GEL compared to VOL (*p* = 0.05), overall.

**TABLE 1:** Physiological variables of eight healthy beagle dogs undergoing colloid fluid infusion for 120 min with 10 mL/kg/h of Gelofusine^®^ (succinylated gelatin) or Voluven^®^ (tetrastarch 130/0.4).

Parameter	Treat	0			15			30			45			60			75			90			105			120		
		Mean	Min	Max	Mean	Min	Max	Mean	Min	Max	Mean	Min	Max	Mean	Min	Max	Mean	Min	Max	Mean	Min	Max	Mean	Min	Max	Mean	Min	Max
HR (bepm)	GEL	79	50	122	91	73	109	90	73	108	89	67	102	91	66	105	90	65	104	92	69	106	94	68	102	94	68	112
	VOL	84	55	103	90	65	109	94	68	109	96	77	109	96	77	107	97	85	107	95	75	105	94	75	107	93	73	105
SAP (mmHg)	GEL	118	98	132	105	80	125	103	90	123	108	93	120	109	97	127	110	92	122	116	92	128	118	97	129	118	90	105
	VOL	120	85	156	116	85	141	116	78	158	113	78	147	113	81	147	112	88	157	111	87	155	115	90	152	111	83	148
MAP (mmHg)	GEL	90	72	110	81	68	98	78	68	89	82	71	94	81	67	94	83	75	94	83	75	94	83	74	94	85	75	94
	VOL	89	70	130	87	70	129	84	68	126	83	70	119	82	69	124	80	64	122	79	67	120	80	65	120	79	64	119
DAP (mmHg)	GEL	74	52	102	67	53	93	67	53	78	68	56	81	67	56	80	69	59	80	67	59	79	68	59	79	70	59	82
	VOL	75	59	111	72	57	117	70	54	110	69	55	107	68	55	109	65	52	105	66	50	104	64	49	103	65	49	103
PP (mmHg)	GEL	44	26	54	35	11	50	36	16	55	39	20	61	42	25	59	42	24	60	49	24	60	48	34	62	48	21	74
	VOL	45	21	66	44	23	65	46	18	65	43	15	69	45	16	74	46	27	68	45	26	67	51	35	70	47	29	62
Temp (°C)	GEL	36.2	35.0	36.9	36.1	35.4	36.9	36.0	34.8	37.2	36.0	35.0	37.4	36.3	35.2	37.5	36.5	35.5	37.6	36.7	35.8	37.8	36.8	36.0	37.7	36.9	36.1	37.8
	VOL	36.2	34.8	37.0	36.0	34.6	36.9	35.9	34.6	36.8	35.9	34.6	37.1	35.9	34.6	37.2	36.0	34.6	37.1	36.0	34.5	37.4	36.0	34.6	37.4	36.1	34.6	37.4
*f*R (brpm)	GEL	8	5	14	9	4	19	10	5	15	10	6	16	11	6	15	13*	6	24	17*	6	36	18*	6	38	17*	9	32
	VOL	10	7	16	11	7	18	11	7	17	11	6	14	11	8	15	11	8	15	13	8	21	12	7	19	12	8	15
PE’CO_2_ (mmHg)	GEL	48	46	53	49	45	56	49	45	54	47	36	57	49	44	57	49	43	60	48	43	63	47	43	59	46	42	54
	VOL	49	44	59	49	44	63	50	43	60	50	44	58	49	43	57	51	43	58	50	43	59	49	44	58	49	44	56
SpO_2_ (%)	GEL	98	93	100	99	96	100	99	95	100	98	95	100	98	95	100	98	94	100	98	95	100	99	94	100	99	94	100
	VOL	100	98	100	99	93	100	99	95	100	99	94	100	99	94	100	99	95	100	99	95	100	99	96	100	99	98	100
FE’iso (%)	GEL	1.3	0.9	1.6	1.4	1.1	1.6	1.3	1.1	1.6	1.4	1.1	1.8	1.3	1.2	1.7	1.3	1.2	1.6	1.4	1.2	1.8	1.4	1.2	1.7	1.3	1.2	1.5
	VOL	1.3	1.1	1.5	1.4	1.2	1.6	1.4	1.3	1.5	1.4	1.3	1.5	1.3	1.3	1.4	1.3	1.2	1.4	1.3	1.2	1.5	1.4	1.2	1.5	1.4	1.2	1.5

Treat, treatment; GEL, Gelofusine^®^; VOL, Voluven^®^; Min, minimum; Max, maximum: HR, heart rate; bepm, beats/minute; SAP, systolic arterial blood pressure; MAP, mean arterial blood pressure; DAP, diastolic arterial blood pressure; PP, pulse pressure; Temp, temperature; *f*R, respiratory rate; brpm, breaths/minute; PE’CO_2_, end tidal carbon dioxide; SpO_2,_ oxygen-haemoglobin saturation; FE’iso, end tidal isoflurane.

Variables are reported as mean values and range from immediately prior to initiation of infusion (time 0 min) for 120 min of infusion.

* , *p* < 0.05 significant finding over time.

There was no effect, between treatments or over time, on blood pH, partial pressure of carbon dioxide, bicarbonate ion concentration and base excess (Table 2). However, the electrolytes potassium (*p* < 0.01) and calcium (*p* < 0.01) did demonstrate an increasing and decreasing difference over time, respectively, with no differences between treatments. However, all measured electrolyte concentrations remained within acceptable clinical ranges.

**TABLE 2:** Measured and calculated blood gas, electrolyte and pH variables of eight healthy beagle dogs undergoing colloid fluid infusion for 120 min with 10 mL/kg/h of Gelofusine^®^ (succinylated gelatin) or Voluven^®^ (tetrastarch 130/0.4).

Parameter	Treat	0			60			120			Ref interval	
		Mean	Min	Max	Mean	Min	Max	Mean	Min	Max	Min	Max
Sodium (mmol/L)	GEL	147	144	156	147	144	154	145	142	151	142	151
	VOL	147	144	150	145	143	149	145	143	148	-	-
Potassium (mmol/L)	GEL	3.9*	3.7	4.0	4.2*	3.5	5.2	4.7*	3.9	6.2	3.6	5.1
	VOL	4.0*	3.5	4.3	4.2*	3.8	4.4	4.5*	4.2	5.2	-	-
Chloride (mmol/L)	GEL	114	112	119	114	112	118	114	112	118	107	117
	VOL	113	110	115	114	111	116	114	112	116	-	-
Ionised calcium (mmol/L)	GEL	1.3	1.2	1.3	1.2	1.	1.3	1.3	1.2	1.3	-	-
	VOL	1.3	1.3	1.3	1.2	1.2	1.3	1.3	1.2	1.3	-	-
PaO_2_ (mmHg)	GEL	472	383	525	516	459	551	496	458	568	-	-
	VOL	447	268	528	482	451	513	486	438	513	-	-
O_2_ content (mmol/L)	GEL	25.8*	25.0	27.0	22.7*	20.0	26.0	21.0*	19.0	25.0	-	-
	VOL	24.5*	19.0	28.0	22.8*	19.0	27.0	22.1*	19.0	26.0	-	-
SaO_2_ (%)	GEL	99.8	99.8	99.9	99.8	99.7	99.9	99.8	99.7	99.9	-	-
	VOL	99.8	99.7	99.9	99.8	99.7	99.9	99.8	99.7	99.9	-	-
PaCO_2_ (mmHg)	GEL	53	48	60	52	42	67	47	37	56	-	-
	VOL	50	45	60	52	45	62	50	43	61	-	-
CO_2_ content (mmol/L)	GEL	26.6	24.2	28.6	26.2	23.0	30.1	25.6	21.1	29.0	-	-
	VOL	26.1	21.0	30.1	26.2	21.8	29.3	26.3	21.9	29.7	-	-
pH	GEL	7.29	7.24	7.33	7.29	7.24	7.35	7.33	7.30	7.36	-	-
	VOL	7.31	7.25	7.40	7.29	7.23	7.40	7.31	7.24	7.42	-	-
HCO_3_^-^ (actual) (mEq/L)	GEL	25.0	22.7	26.9	24.5	21.5	28.1	24.0	19.9	27.2	-	-
	VOL	24.5	19.6	28.3	24.6	20.2	27.9	24.7	20.4	27.8	-	-
HCO_3_^-^ (standard) (mEq/L)	GEL	22.3	20.7	23.5	22.1	19.7	24.2	22.6	20.2	24.4	-	-
	VOL	22.3	18.6	26.5	22.2	18.2	26.7	22.5	18.5	26.9	-	-
BE (ECF) (mEq/L)	GEL	−1.7	−4.3	0.3	−2.2	−5.7	0.7	−2.1	−5.9	0.9	-	-
	VOL	−2.0	−7.6	2.7	−2.1	−7.6	3.0	−1.8	−7.3	3.0	-	-

Treat, treatment; GEL, Gelofusine^®^; VOL, Voluven^®^; Min, minimum; Max, maximum; PaO_2,_ partial pressure of oxygen; SaO_2,_ arterial oxygen saturation; PaCO_2_, partial pressure of carbon dioxide; HCO_3_^-^, bicarbonate ion concentration; BE (ECF), base excess extra cellular fluid.

Variables are reported as mean values and range from immediately prior to initiation of infusion (time 0 min) for 120 min of infusion.

* , *p* < 0.05 significant finding over time.

The erythrogram demonstrated changes over time, but not between the treatments, where the haemoglobin concentration, erythrocyte count and haematocrit decreased significantly over time but remained within normal clinical ranges (all *p* < 0.01; Table 3). The erythrocyte morphology (mean corpuscular volume, haemoglobin concentration and distribution width) did not differ between treatments or over time. The leukogram demonstrated a similar change over time, where the total leucocyte count (*p* = 0.01) and segmented neutrophil count (*p* = 0.04) decreased over time but did not differ between treatments. The other differential leukocyte counts were also not different between groups or over time and were within normal clinical ranges. The thombogram demonstrated a significant difference between GEL (250 [83, 371] × 10^9^/L) and VOL (343 [171, 526] × 10^9^/L) (*p* = 0.02), but not over time.

**TABLE 3:** Haematological variables of eight healthy beagle dogs undergoing colloid fluid infusion for 120 min with 10 mL/kg/h of Gelofusine^®^ (succinylated gelatin) or Voluven^®^ (tetrastarch 130/0.4). Variables are reported as mean and range from immediately prior to initiation of infusion (time 0 min) and at the end of infusion (time 120 min).

Parameter	Treat	0			120			Ref interval	
		Mean	Min	Max	Mean	Min	Max	Min	Max
Haemoglobin (g/L)	GEL	168*	144	191	140*	128	160	120	180
	VOL	162*	128	186	143*	124	164	-	-
Erythrocyte count (x 10^12^/L)	GEL	7.2*	6.0	8.0	6.0*	5.4	7.2	5.5	8.5
	VOL	7.1*	5.9	8.5	6.2*	5.6	7.5	-	-
Haematocrit (L/L)	GEL	0.50*	0.42	0.56	0.42*	0.38	0.49	0.37	0.55
	VOL	0.49*	0.39	0.58	0.43*	0.37	0.51	-	-
Mean corpuscular volume (fL)	GEL	69.7	67.1	71.3	69.8	66.8	71.6	60	77
	VOL	69.3	66.2	73.0	69.3	66.0	72.3	-	-
Mean corpuscular haemoglobin (pg)	GEL	23.3	21.8	24.5	23.4	22.0	24.2	-	-
	VOL	22.9	21.7	25.1	23.0	21.8	24.5		-
Mean corpuscular haemoglobin concentration (g/dL)	GEL	33.4	32.5	34.5	33.5	32.6	34.1	32	36
	VOL	33.1	31.7	34.4	33.2	32.0	34.4	-	-
Red cell distribution width (%)	GEL	12.9	11.9	14.0	12.9	12.0	13.9	-	-
	VOL	12.9	12.2	13.8	12.8	12.2	13.7	-	-
Leukocyte count (×10^9^/L)	GEL	6.5*	5.6	8.6	5.1*	3.4	6.3	6	15
	VOL	7.1*	4.9	8.8	5.7*	2.6	9.5	-	-
Neutrophil (Segmented) (×10^9^/L)	GEL	4.4*	2.8	6.4	3.3*	2.3	4.7	3	11.5
	VOL	4.6*	3.3	6.9	3.9*	2.6	6.1	-	-
Neutrophil (Band) (×10^9^/L)	GEL	0.02	0.00	0.12	0.00	0.00	0.03	0	0.5
	VOL	0.00	0.00	0.00	0.02	0.00	0.09	-	-
Lymphocyte (×10^9^/L)	GEL	1.4	1.0	1.8	1.2	0.7	1.8	1	4.8
	VOL	1.7	1.1	2.4	1.5	0.8	0.8	-	-
Monocyte (×10^9^/L)	GEL	0.28	0.06	0.49	0.22	0.05	0.42	0.15	1.35
	VOL	0.33	0.08	0.70	0.21	0.09	0.47	-	-
Eosinophil (×10^9^/L)	GEL	0.50	0.28	0.89	0.37	0.09	1.28	0.1	1.25
	VOL	0.40	0.18	0.75	0.26	0.14	0.39	-	-
Basophil (×10^9^/L)	GEL	0.00	0.00	0.00	0.00	0.00	0.00	0	0.1
	VOL	0.00	0.00	0.00	0.00	0.00	0.00	-	-
Thrombocyte count (×10^9^/L)	GEL	263	83	371	238	181	299	200	500
	VOL	346	171	526	341	187	511	-	-

Treat, treatment; GEL, Gelofusine; VOL, Voluven; Min, minimum; Max, maximum; Ref, reference.

* , *p* < 0.05 significant finding over time.

There were no differences between treatments or over time for the TEG, aPTT and PT tests of haemostasis (Table 4). Also, all of the parameters in each test were within normal clinical ranges.

**TABLE 4:** Thromoboelastography and coagulation indicators of eight healthy beagle dogs undergoing colloid fluid infusion for 120 min with 10 mL/kg/h of Gelofusine^®^ (succinylated gelatin) or Voluven^®^ (tetrastarch 130/0.4).

Parameter	Treat	0			120			Ref intervals	
		Mean	Min	Max	Mean	Min	Max	Min	Max
*R*-time (minutes)	GEL	3.3	3.0	3.9	2.9	2.0	3.8	1.8	7.3
	VOL	3.2	2.6	4.0	5.5	2.1	22.3	-	-
K-time (minutes)	GEL	1.9	1.3	3.3	2.0	1.3	2.9	1	3.6
	VOL	1.6	0.8	2.8	1.5	0.0	3.5	-	
Angle (degrees)	GEL	65.2	54.4	70.0	63.3	54.8	71.0	49.1	74.5
	VOL	68.3	56.6	77.9	59.7	5.2	78.9	-	-
MA (mm)	GEL	57.4	46.0	64.5	54.8	49.4	63.9	48.9	74.3
	VOL	58.7	48.1	70.0	54.3	11.4	75.0	-	-
G-value (dyn/cm)	GEL	6.9	4.3	9.1	5.8	2.6	8.9	4.7	14.4
	VOL	7.4	4.6	11.7	7.4	0.6	15.0	-	
Lysis 30 (%)	GEL	0.35	0.00	2.80	0.00	0.00	0.00	0	2.4
	VOL	0.30	0.00	2.30	0.00	0.00	0.00	-	-
Lysis 60 (%)	GEL	0.76	0.00	4.60	0.08	0.00	0.50	0	8
	VOL	1.89	0.10	9.30	0.58	0.00	1.60	-	-
aPTT (seconds)	GEL	12.0	10.4	15.5	12.2	10.3	16.5	10.2	18.1
	VOL	12.1	10.4	15.0	13.4	10.3	20.2	-	-
PT (seconds)	GEL	15.4	7.4	38.9	14.4	7.3	33.0	10.2	18.1
	VOL	16.0	7.4	45.4	13.8	7.7	34.6	-	

Treat, treatment; GEL, Gelofusine^®^; VOL, Voluven^®^; Min: minimum; Max, maximum; Ref, reference; aPTT, activated partial thromboplastin time; PT, prothrombin time; MA, maximum amplitude.

Variables are reported as mean value and range from immediately prior to initiation of infusion (time 0 min) and at the end of infusion (time 120 min).

## Discussion

The constant infusion rate of either VOL or GEL, at 10 mL/kg/h for 120 min, to normovolaemic dogs did not cause any clinically relevant alterations in acid–base, haematological or haemostatic effects.

Although the half-life of VOL is not known in dogs, in humans, the distribution half-life has been reported to be 1.39 h (Waitzinger et al. 1998). Another study (Lobo et al. 2010) has found only 21% and 16% of GEL and VOL, respectively, had escaped from the vasculature after 1 h in humans. Thus, unless the pharmacokinetic profile is drastically different in dogs, it is hypothesised that a significant proportion of the total fluids administered would have been present in the vasculature at 120 min and would have been available to affect coagulation. This effect is evident as significant dilution of the haemogram was present at 120 min. As no blood was lost during the procedure, besides the small volume taken for sample collection, it is inferred that there was a linear relationship between the fractional decrease in haematocrit and gain in blood volume. This gain in blood volume over 120 min was estimated using the following formula (Gross 1983; Equation 2):

$$Vl=\text{EBV}\frac{Ho-Hf}{Ho}$$ [Eqn 2]

where *Vl* is the blood volume gained, EBV is the estimated blood volume, *Ho* is the initial haematocrit and *Hf* is the final haematocrit (Gross 1983). Estimating the blood volume of dogs to be 85 mL/kg, we concluded that the average circulating volume gain by 120 min was 13.6 mL/kg and 10.4 mL/kg for GEL and VOL respectively. These values allow comparison of results from this study with other studies administering boluses of fluid or using *in vitro* dilutions. These values provide some indication of the circulating half-life of these colloids, which appear to be very similar to those reported in humans. It also demonstrates that the volume expanding capabilities of both colloids are similar. This is illustrated by a significant decrease in the haematocrit, haemoglobin concentration, erythrocyte count and leukocyte count over time with no differences between the groups. This is in agreement with studies in humans which showed that GEL and VOL (12.5 mL/kg – 15 mL/kg over 1 h) resulted in similar blood volume expansion (Lobo et al. 2010).

In this study, the effects of the two colloids on coagulation were evaluated using TEG and routine PT and aPTT. A combination of these tests allowed evaluation of both the enzymatic steps involved in the coagulation process and the global coagulation from the beginning of clot formation to fibrinolysis, including the effects of cellular components and cell membranes on coagulation (McMichael & Smith 2011). In the current study, 10 mL/kg/h (total dose of 20 mL/kg) of either colloid did not produce significant changes in coagulation parameters in comparison to each other or to baseline after 120 min. In contrast to this study, *in vitro* dilution of canine whole blood with HES 130/0.4 at a ratio of 1:10, 1:5.5 and 1:4 (simulating *in vivo* dosages of 10 mL/kg, 20 mL/kg and 30 mL/kg respectively) showed dose-dependent hypocoagulability on viscoelastic profiles (Griego-Valles et al. 2017; McBride et al. 2013). Similarly, *in vitro* and *in vivo* haemodilution of human whole blood with GEL has produced hypocoagulable profiles at dilutions as low as 10% – 20% and 15 mL/kg respectively (Brazil & Coats 2000; Niemi et al. 2006). As this study infused the fluids over 120 min, it is possible that the dose and infusion rate were too low for changes to be detected. However, the doses used were chosen based on current clinical recommendations of the American Animal Hospital Association for anesthetised patients (Davis et al. 2013). The absence of changes suggests that GEL and VOL could be used at recommended doses without coagulation deficits being expected in healthy animals when administered as a 120-min infusion.

The maintenance of blood pH within a narrow range is essential to preserve normal physiological functioning of organ systems. Deviations could manifest as altered cardiac contractility, arrhythmias, hypotension, diminished responsiveness to catecholamines, insulin resistance, dysfunction of enzyme systems and electrolyte disturbances (Kuleš et al. 2015; Moning 2013).

In this study, no major changes were found in acid–base parameters. This observation is in contrast to a similar study, where 15 mL/kg HES 130/0.42 suspended in saline and administered over 30 min – 40 min resulted in a significant alteration in blood pH because of increased chloride, decreased bicarbonate and SID (Adamik, Obrador & Howard 2018). A major difference between that study and the current study was the administration of fluids as a bolus versus a 120-min infusion. These differences highlight the importance of fluid dosage per time in causing alterations in blood pH with fluids administered rapidly being more likely to cause alterations than the same dose administered over a longer period. Furthermore, a shift in chloride ion did not occur in our dogs and therefore no hyperchloraemic acidosis, as described previously, was detected (Langer et al. 2014; Muir 2017).

The overall lack of clinically relevant changes detected by this study could be attributed to the sample size. However, statistically significant findings were detected and other studies investigating the acid–base and haemostatic effects recruited a similar number of dogs as those enrolled in our study (Falco et al. 2012; McBride et al. 2013, 2016). Therefore, it was concluded that the sample size of this randomised crossover study was sufficient to draw clinically relevant conclusions. A limitation to the study was that all the complex processes responsible for acid–base changes (Stewart calculations) and haemostatic effects (flow cytometry, platelet closure time, activity and concentration of individual clotting factors) were not investigated. Therefore, comment cannot be made on the effects these colloid fluids may have had on all potential contributing parameters. Another limitation is that a total dose of 20 mL/kg was administered, which may not have been a large enough dose to unmask changes of clinical relevance. However, this dose was selected based on the currently recommended maximum ‘safe’ dose to treat hypovolaemia in dogs (Davis et al. 2013). Furthermore, the aim was not to volume overload normovolaemic dogs, and thus we kept the study’s total dose at 20 mL/kg. In addition, a dose of 20 mL/kg could cause significant changes in hypovolaemic dogs but not in normovolaemic dogs, so further studies in such animals are warranted.

Finally, the role of dosage per time is important. Most documented cases of derangements occurring are during studies using dosage rates over 70 mL/kg/h (West et al. 2013). Such an infusion rate, administered over 120 min should yield a final haematocrit of 0.29 using Gross’ equation (assuming an initial haematocrit of 0.5 and that 35 mL/kg of the colloid remains in the vasculature after 120 min; Gross 1983). This is still more than the critical haematocrit suggested in literature at which oxygen demand equates oxygen delivery (Champion, Periera Neto & Camacho 2011; Crystal 2015). It is therefore suggested that the future haemodilution studies explore dosage rates of between 10 mL/kg/h and 70 mL/kg/h to determine optimal administration guidelines.

## Conclusion

This study demonstrates that GEL and VOL have similar blood volume expanding effects and circulating half-lives in dogs. At doses of 10 mL/kg/h administered over 120 min, GEL and VOL have comparably negligible effects on acid–base balance, haematology and coagulation in normovolaemic dogs.
